# Unlocking prognostic potential: A genomic signature of caloric restriction in patients with epithelial ovarian cancer

**DOI:** 10.1371/journal.pone.0317502

**Published:** 2025-01-16

**Authors:** Ori Tal, Tamar Zahavi, Liat Anabel Sinberger, Mali Salmon-Divon

**Affiliations:** 1 Department of Obstetrics and Gynecology, Division of Gynecologic Oncology, Edith Wolfson Medical Center, Holon, Israel; 2 Faculty of Medicine, Tel-Aviv University, Tel Aviv-Yafo, Israel; 3 Faculty of Natural Sciences, Department of Molecular Biology, Ariel University, Ariel, Israel; 4 Adelson School of Medicine, Ariel University, Ariel, Israel; Instituto do Cancer do Estado de Sao Paulo / University of Sao Paulo, BRAZIL

## Abstract

**Objectives:**

Epithelial ovarian cancer is a significant contributor to cancer-related mortality in women, frequently recurring post-treatment, often accompanied by chemotherapy resistance. Dietary interventions have demonstrated influence on cancer progression; for instance, caloric restriction has exhibited tumor growth reduction and enhanced survival in animal cancer models. In this study, we calculated a transcriptomic signature based on caloric-restriction for ovarian cancer patients and explored its correlation with ovarian cancer progression.

**Methods:**

We conducted a literature search to identify proteins modulated by fasting, intermittent fasting or prolonged caloric restriction in human females. Based on the gene expression of these proteins, we calculated a Non-Fasting Genomic Signature score for each ovarian cancer sample sourced from the Cancer Genome Atlas (TCGA) database. Subsequently, we examined the association between this genomic profile and various clinical characteristics.

**Results:**

The non-fasting genomic signature, comprising eight genes, demonstrated higher prevalence in primary ovarian tumors compared to normal tissue. Patients with elevated signature expression exhibited reduced overall survival and increased lymphatic invasion. The mesenchymal subtype, associated with chemotherapy resistance, displayed the highest signature expression. Multivariate analysis suggested the non-fasting genomic signature as a potential independent prognostic factor.

**Conclusions:**

Ovarian cancer tumors expressing a “non-fasting” transcriptional profile correlate with poorer outcomes, emphasizing the potential impact of caloric restriction in improving patient survival and treatment response. Further investigations, including clinical trials, are warranted to validate these findings and explore the broader applicability of non-fasting genomic signatures in other cancer types.

## Introduction

Epithelial ovarian cancer ranks as the seventh most prevalent cancer in women, and stands as the eighth leading cause of female mortality worldwide [1]. About 75% of women diagnosed with ovarian cancer present with advanced disease, characterized by International Federation of Gynecology and Obstetrics (FIGO) stage IIIC or beyond [2]. Patients are treated by surgery followed by chemotherapy, or neoadjuvant chemotherapy with interval cytoreductive surgery [3], with an ultimate goal of achieving optimal cytoreduction [4]. Although this yields complete remission in 60–80% of patients, recurrent disease surfaces in nearly 80% of patients, often with chemotherapy resistance [5].

Therefore, research has directed much effort towards enhancing chemotherapy efficacy while minimizing resistance. To this end, the emergence of next generation sequencing, and specifically RNA (ribonucleic acid) sequencing and transcriptomics, has been increasingly employed in an effort to discover possible mechanisms behind chemotherapy resistance [6]. Transcriptomics analyses were performed in patients with recurrent ovarian cancer with and without chemotherapy resistance, revealing distinct transcriptomic profiles in progressive tumors [7]. Other studies examining pre- and post-chemotherapy patient tissues have identified elevated expression of stress response genes [8].

Nevertheless, one aspect of transcriptomics remains understudied. One of the hallmarks of cancer is its sensitivity to reduced nutrients in its environment. Indeed, many studies have shown caloric restriction creates environments which are hostile to cancer growth, diminishing its ability to adapt and survive [9]. This nutritional deficit proves advantageous in chemotherapy treatment, since it has the double potential of reducing chemotherapy toxicity and increasing their efficacy, by modulating growth factors and metabolites [9]. Nonetheless, clear dietary guidelines for ovarian cancer patients, including caloric restriction as a supplementary approach, remain absent. In this study, we sought to assess whether ovarian cancers manifest a transcriptomic susceptibility to caloric restriction, and to examine its impact on patient outcomes.

## Materials and methods

### Development of a “non-fasting” gene signature

To delineate the genomic profile associated with caloric restriction in ovarian cancer patients, we conducted an extensive review of literature from PubMed and Google Scholar. Our aim was to identify proteins exhibiting altered expression patterns during caloric restriction in human females. We selected two comprehensive reviews and one research article that delve into how caloric restriction influences gene expression and specific molecular pathways [10–12]. Given the diverse range of tissues studied in these investigations, we then focused on the most frequently mentioned proteins. We cross-referenced our findings with studies specifically addressing the response of ovarian tissue to caloric restriction. Due to the scarcity of trials conducted on human ovarian tissue, we also incorporated data from non-human primates and mice [13–16].

**Fig 1:**
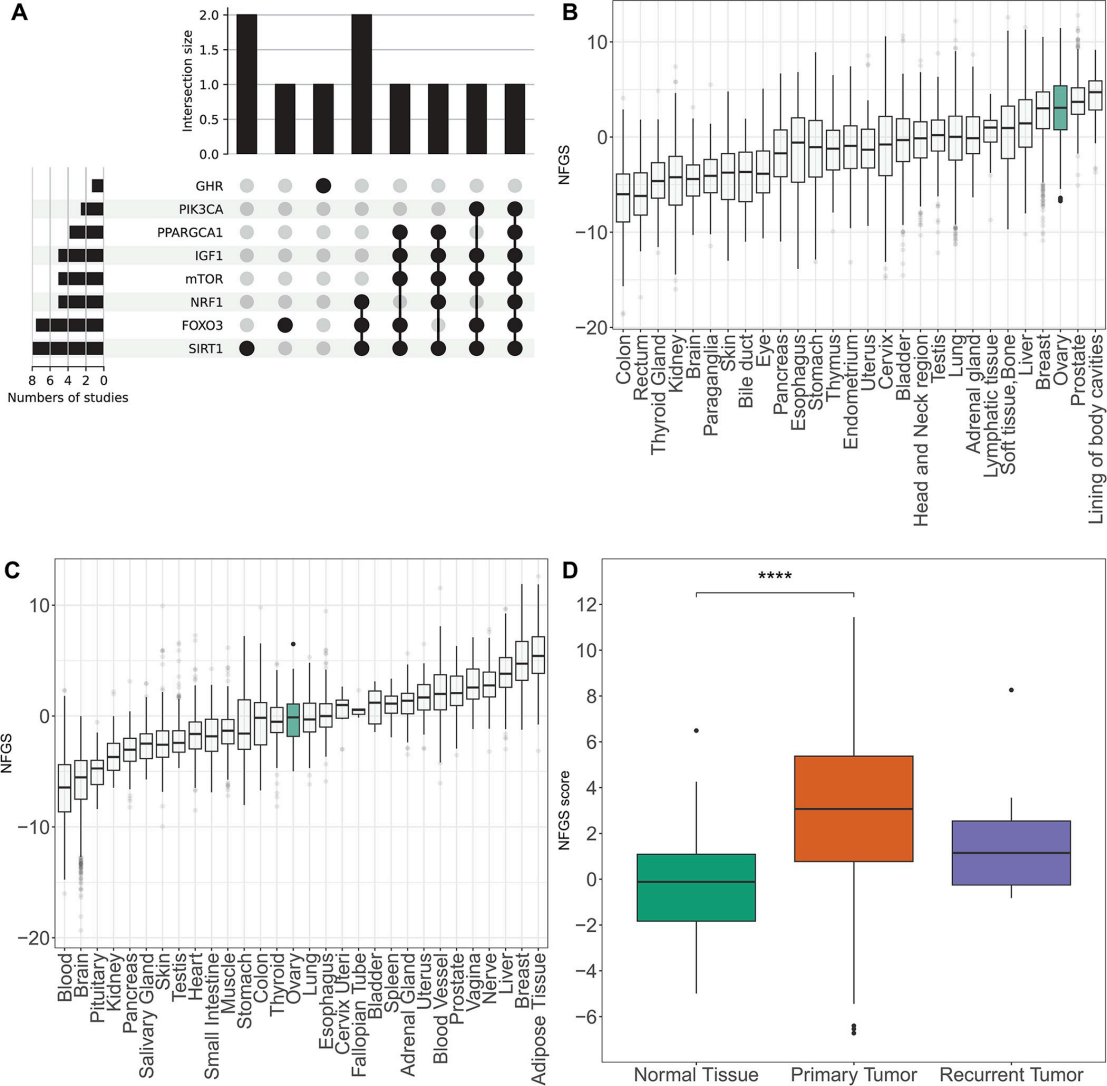
The non-fasting genomic signature is more prevalent in primary ovarian tumors compared with normal tissue. (A) UpSet plot showing the number of unique and shared proteins between eight different studies. Left- Graph shows the total number of studies in which the protein appeared. Right-Intersection of sets of genes at multiple studies. Each column corresponds to a study or set of studies (dots connected by lines below the X axis) containing the same proteins. (B-C) Distribution of the non-fasting genomic signature in (B) 28 types of primary tumors; ovarian tumor is highlighted in green (C) 30 types of normal tissues; ovarian tissue is highlighted in green (D) Distribution of the non-fasting genomic signature in ovarian normal tissue (n = 88), primary tumor (n = 419) and recurrent tumor (n = 8); **** = p <0.0001, Wilcoxon rank sum test.

Based on this analysis, we developed a genomic signature termed the "Non-Fasting Genomic Signature” (NFGS), which reflects the transcriptional landscape of ovarian tissue under a "non-fasting" state, in order to explore the baseline gene expression patterns in ovarian tissue and gain insights into its typical molecular state. From the extensive list of proteins reported in the selected studies, we prioritized those that were most frequently cited or identified as critical regulators in the context of metabolic signaling (Fig 1A). Our final selection included four downregulated proteins (SIRT1, FOXO3, NRF1, and PPARGC1A) and four upregulated proteins (IGF-1, GHR, mTOR, and PIK3CA) characteristic of the "non-fasting" state.

We used this gene expression profile to calculate a signature score for each patient [17]. We computed the sum of the products of the gene coefficient (−1 or 1, depending upon downregulation or upregulation, respectively) by the corresponding normalized gene expression value as described in the following formula where e_i_ is the normalized gene expression value and c_i_ is the gene coefficient:

$$Non-fasting\;genomic\;signature\;=\sum_{i\;=\;1}^{8}e_{i}*c_{i}$$

### Genomic data collection

Employing the Xena Genome Browser by University of California, Santa Cruz, we applied the generated genomic signature in various analyses. Initially, within the combined cohort of the Cancer Genome Atlas (TCGA) Target GTEx (Genotype-Tissue Expression) databases [18], we restricted our focus to ovarian tissue, comparing the prevalence of the non-fasting genomic signature expression profile calculated according to the “RSEM norm_count” expression dataset, across normal ovarian tissue, primary tumor and recurrent tumors. Subsequently, we accessed the TCGA Ovarian Cancer database and extracted gene expression array AffyU133a dataset. The genomic signature was utilized for the assessment of survival data (specifically of stage IIIC and IV tumor patients), lymphatic invasion and gene expression, all available through the Xena genome browser.

To calculate the distribution of the non-fasting genomic signature gene signature across 30 normal tissue types and 28 different primary tumor tissues, we used the downloaded GTEx and TCGA gene expression data. Signature metagene score was calculated for each patient as described above, and the distribution of those scores across normal and tumor samples was visualized using boxplots. For survival analysis, patients were divided into two groups, those with high and those with low signature scores, using the median as the dichotomous cutoff.

### Statistical analysis

Distributions of the non-fasting genomic signature scores were compared with Wilcoxon rank sum test between the control group and the groups of ovary cancer patients. The nonparametric Kruskal-Wallis test followed by the Dunn post hoc test were performed to compare continuous parameters between different groups. Survival curves were estimated using the Kaplan–Meier method and visualized by Survival (Version 3.5–3) and Survminer (Version 0.4.9) R packages, and p-values were calculated using the log-rank test. A multivariate linear regression analysis was carried out to test for a correlation between the non-fasting genomic signature scores and the clinical features. Effects were considered statistically significant at a two-sided P < 0.05. All statistical analyses were performed using the R statistical framework (v.4.4.1). A ggplot2 R package (Version 3.3.5) was used for generating plots.

## Results

### The non-fasting genomic signature is more prevalent in primary ovarian tumors compared with normal tissue

The characteristics of the proteins constituting the non-fasting genomic signature are summarized in Table 1. The results of the literature search are presented as an UpSet plot to visualize the shared proteins between different studies (Fig 1A).

**Table 1:** Proteins linked to caloric restriction, whose gene expression was used in the calculation of non-fasting signature scores.

Protein	Description	Up / Down -regulated under a “non-fasting” state
SIRT1	NAD-dependent deacetylase that plays a key role in multiple biological processes, including cellular senescence, apoptosis, sugar metabolism, inflammation, and fatty liver diseases	downregulated
FOXO3	Transcription factor that regulates genes involved in stress resistance, metabolism, cell cycle arrest, and autophagy	downregulated
NRF1	Transcription factor that maintains cellular homeostasis, embryonic development, and mitochondrial homeostasis; directly regulates PGC1α	downregulated
PPARGC1A	Transcriptional coactivator which has been linked to the pathogenesis of type 2 diabetes and is involved in the antioxidant response upon mild redox and metabolic imbalance	downregulated
IGF-1	A growth hormone that mediates the anabolic and linear growth promoting effects of growth hormone, while independently affecting tissue growth and development, proliferation and lipid metabolism	upregulated
GHR	Mediates the effects of growth hormone (GH) by activating the JAK-STAT signaling pathway, leading to the regulation of various physiological processes such as growth, metabolism, and immune function	upregulated
mTOR	Protein kinase that plays a crucial role in regulating cell growth, survival, metabolism, and immunity, acting as a master regulator of a cell’s growth and metabolic state in response to nutrients and growth factors	upregulated
PIK3CA	Encodes for a subunit of an enzyme called phosphatidylinositol 3-kinase (PI3K), which plays a role in mediating cell survival, differentiation, and proliferation, and has been linked to the development of cancer	upregulated

Distribution of the signature was evaluated in 28 types of primary tumors. It showed the highest median in prostate and ovarian cancers, while colon and rectum tumors showed the lowest median expression (Fig 1B). Different results were obtained in analyzing the distribution of the signature in normal tissues (Fig 1C). Focusing our study on ovarian cancer, the distribution of the signature score was evaluated in tumors of the ovary. A total of 515 ovarian tissue samples were included in this query: 88 normal tissue samples sourced from the GTEx database along with 419 primary tumor samples and 8 recurrent tumor samples both from the TCGA database. Fig 1D illustrates the distribution of the non-fasting genomic signature score across these distinct tissue types. The signature exhibited a notably lower presence within the normal tissue cohort in comparison to tumor tissue (p<0.001, median values of: -0.1, 3.07 and 1.15 for normal tissue, primary tumor and recurrent tumor, respectively). The expression of each gene contributing to the signature score was individually assessed in ovarian cancer tissue compared to controls (S1 Fig). IGF1 and mTOR were upregulated in the tumor tissue, no significant differences in PIK3CA gene expression were demonstrated and the rest were significantly downregulated. Finally, we compared the non-fasting genomic signature score in primary tumors vs. normal samples in different cancers. As demonstrated in S2 Fig, testis and prostate tumors behave similarly to ovarian cancer, while colon and breast tumors demonstrate an opposite trend.

### The non-fasting genomic signature is associated with different clinicopathological characteristics

**Fig 2:**
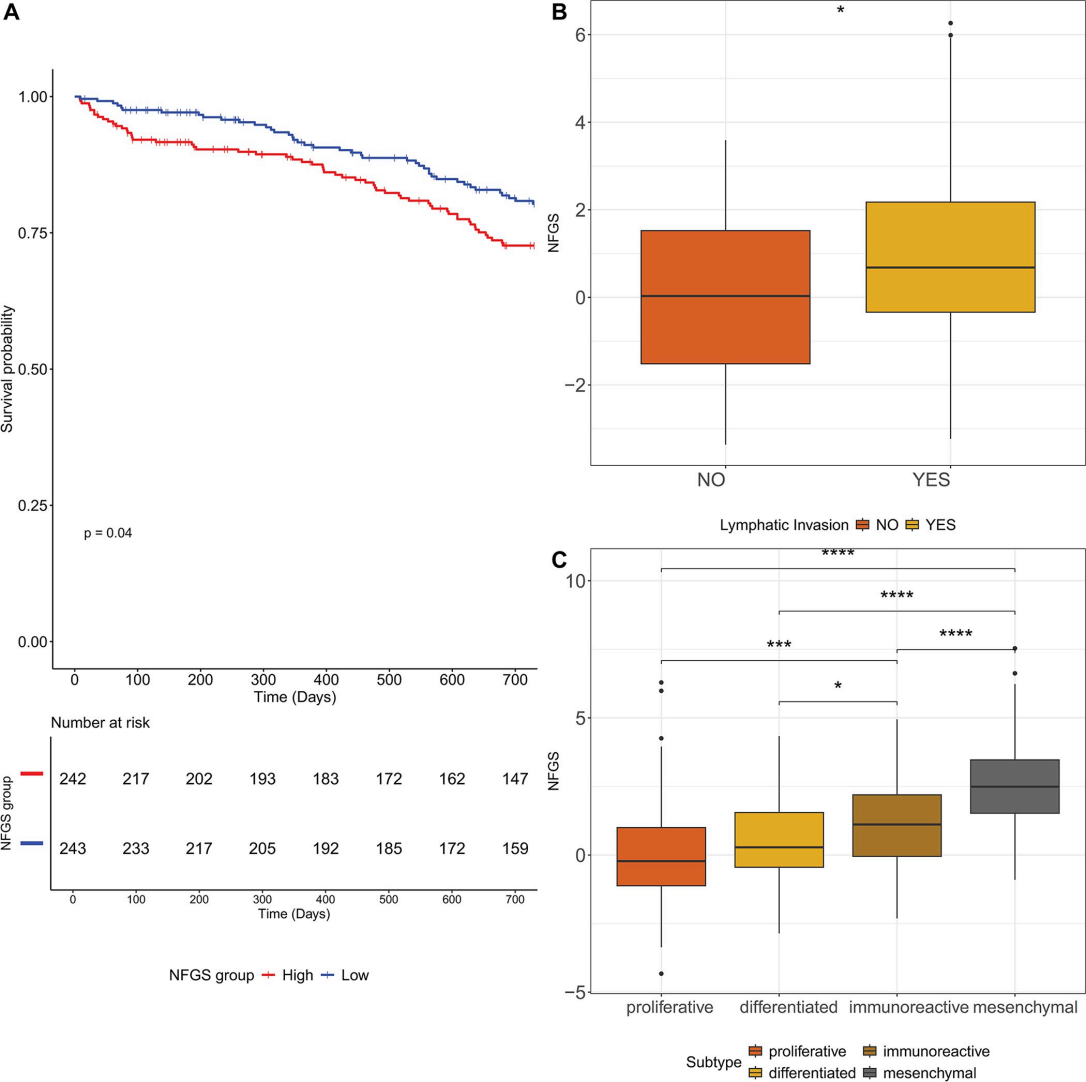
(A) Kaplan-Meier survival curve illustrating the overall survival comparison between non-fasting genomic signature overexpression (depicted in red) and underexpression (depicted in blue) across a span of 730 days (B) Distribution of the non-fasting genomic signature score among patients with or without Lymphatic invasion (n = 137 and n = 80, respectively) (C) Distribution of the non-fasting genomic signature score among the four distinct tumor molecular subtypes: Proliferative (n = 64), differentiated (n = 61), immunoreactive (n = 67) and mesenchymal (n = 66). Wilcoxon rank sum test; *p<0.05, **p<0.01, ***p<0.001, ****p<0.0001.

We conducted Kaplan-Meier analysis to assess overall survival of patients with FIGO stage IIIC or IV (after excluding recurrent tumor samples) based on their non-fasting genomic signature expression. Patients exhibiting the nondeleterious profile (depicted in blue) demonstrated a statistically significant improvement in two-year overall survival (Hazard ratio:1.5, 95% CI: 1.02–2.23, p<0.05) compared to those expressing high signature (Fig 2A). Nevertheless, no statistically significant difference in 5-year overall survival was observed between the groups (data not shown).

Next, we assessed this patient cohort for data on residual disease post-surgery, by comparing patients with no macroscopic residual disease to those with varying degrees of residual disease. Data was available for 457 individuals. No statistically significant differences in the signature were identified between the groups (S3 Fig).

We also sought to examine the relationships between the non-fasting genomic signature and lymph node invasion. Among all stages, information on lymphatic invasion was available for 217 patient samples. Comparative analysis of signature distribution between patients with lymphatic invasion and those without revealed a statistically significant elevation in signature expression among patients with lymphatic invasion (median = 0.681) compared to those without (median = 0.047, p = 0.002, as depicted in Fig 2B).

Subsequently, we assessed signature distribution across the four distinct tumor molecular subtypes of ovarian cancer: proliferative, differentiated, immunoreactive and mesenchymal. Our findings indicate a significant disparity in signature distribution among these subtypes (p<0.0001, Kruskal–Wallis test, Fig 2C). Notably, the mesenchymal group presented the highest expression levels of the non-fasting genomic signature (median = 2.53), followed by immunoreactive (median = 0.995,), differentiated (median = 0.275), and proliferative displaying the lowest expression (median = -0.279). Finally, we used multivariate Cox regression analysis to quantify the effect of the signature on survival after adjusting for age and stage. The signature demonstrated a hazard ratio (HR) of 1.45 with a 95% confidence interval of [0.98,2.16] for the high group compared to the low group, and p value 0.063. Age exhibited a HR of 1.05 (95% CI: [1.03,1.07], p value<0.001). Cancer stage showed a significant impact, with a HR of 1.96 (95% CI: [1.25,3.10], p value = 0.004) for stage 4 compared to stage 3 (S4 Fig).

## Discussion

In this study, we have formulated a transcriptional profile resembling a “non-fasting state”, and discovered that it is extremely prevalent in ovarian cancer. We have shown that ovarian cancer patients overexpressing this signature tend to experience reduced overall survival and increased lymphatic invasion compared to those with a “fasting” state. Lastly, we observed that mesenchymal ovarian tumors, typically associated with poorer prognosis and increased platinum resistance, tend to express “non-fasting” profiles.

The connection between cancer and diet, particularly the Western one, has long been linked to adverse nutritional effects and an elevated risk of various cancers [19]. Consequently, dietary interventions have garnered interest as potential complementary strategies alongside conventional cancer treatments [20]. These interventions encompass diverse methods, including caloric restriction (reducing overall calorie intake while still maintaining adequate nutrient intake), fasting variations, and specialized diets like the ketogenic diet and macronutrient manipulation [21]. While an exhaustive description of each approach is beyond the scope of this article, the common objective is to manipulate tumor metabolism and treatment responsiveness. These approaches have been investigated in animal models [22], and have shown promising results in human subjects as well. Caloric restriction has been shown to render cancer cells more susceptible to chemotherapy, reduce the side effects of cancer treatment, and even prevent development of certain types of cancer [23–25]. As an example, transcriptomic analysis of patients with breast cancer undergoing fasting-mimicking diets alongside standard chemotherapy exhibited enhanced intratumor Th1/cytotoxic responses and an elevation of other immune signatures that are correlated with better outcomes in cancer patients [26].

The transcriptomic profile of caloric restriction has been extensively investigated across various tissues, revealing predominant alterations in inflammation pathways, DNA replication, cell cycle functions and oxidative stress response [11]. A consistent and almost universal outcome observed in ovarian tissue under caloric restriction is the increased expression of SIRT-1 and its downstream targets [16,27]. SIRT-1, a member of NAD+-dependent deacetylases family known as sirtuins, is linked to the upregulation of multiple proteins, including PGC-1α, NRF-1 and FOXO3a [16,28]. Additional pervasive response to caloric restriction in ovarian tissue is a downregulation of growth hormone receptor (GHR), insulin-growth factor 1 (IGF1) and their products [13,14]. The downregulation of these genes leads, via the PI3-AKT pathway, to a reduced expression of mTOR, a protein kinase that promotes cell growth and metabolism [10,12]. Based on this information, we formulated a genomic signature depicting an unfavorable non-fasting state, comprising four upregulated proteins (mTOR, PIK3CA, IGF1 and GHR) and four downregulated proteins (SIRT1, NRF1, FOXO3 and PGC-1α).

Utilizing the TCGA Target GTEx database, we found that ovarian cancer has one of the highest expressions of the non-fasting genomic signature among primary cancers (Fig 1B). Specifically in ovarian tissue, we observed a notably elevated expression of this adverse genomic signature within cancerous ovarian tissue, as compared to normal tissue. However, this did not hold true for all types of cancers; while testicular cancer and prostate cancer adhered to our expected trend, colon and breast cancer displayed an opposite signature (S4 Fig), leading us to hypothesize that the signature we formulated is specific to ovarian cancer, and possibly other cancers of the genital system.

Subsequently, in our analysis of the TCGA Ovarian Cancer dataset, we discovered that among patients at stage IIIC and beyond, those overexpressing the non-fasting genomic signature had a significantly poorer two-year overall survival compared to the underexpressing group. Importantly, in a multivariate analysis, the non-fasting genomic signature showed a trend towards significance in influencing ovarian cancer patient survival (HR = 1.45, p = 0.06), independent of age and cancer stage, underscoring its potential as an additional and independent prognostic factor for ovarian cancer patients. The observed improvement in two-year overall survival, without a corresponding significant improvement in five-year survival, may reflect a short-term benefit that diminishes over time, potentially due to tumor progression, late-emerging treatment effects, or differences in subsequent therapies. Additionally, early survival benefits could be influenced by transient biological mechanisms, such as an initial immune response, that do not sustain long-term survival advantages. Furthermore, across all stages, patients with lymphatic invasion demonstrated a statistically significant higher expression of signature compared to those without it. These trends agree with the aforementioned studies regarding various fasting methods in cancer. In animal models, intermittent fasting has shown a 20% improvement in overall survival in colorectal cancer and a 30% improvement in breast cancer [29]. Fasting has also been shown to improve overall survival when combined with chemotherapy [30], and various forms of caloric restriction have shown promising results in inhibit cancer spread in animals [31]. It is important to acknowledge that the TCGA dataset does not distinguish between high-grade and low-grade serous carcinomas. Nevertheless, because 95% of serous adenocarcinomas are high-grade, our findings are highly representative of high-grade serous carcinoma outcomes [32].

Lastly, we examined our genomic signature among the four distinct TCGA gene expression profile subtypes of ovarian cancer, namely immunoreactive, proliferative, differentiated and mesenchymal subtypes. These subtypes have been found to confer different prognoses, with the immunoreactive presenting the best and the mesenchymal the worst [33]. The non-fasting genomic signature was significantly overexpressed in the mesenchymal group; this was followed by increasingly lower levels in the immunoreactive, differentiated and proliferative groups, respectively. The epithelial-mesenchymal transition, a process by which epithelial cells acquire mesenchymal characteristics such as increased motility and invasion, is associated with the upregulation of genes involved in cell migration and invasion, and is associated with platinum-resistance in epithelial ovarian cancer [34]. Platinum-resistant ovarian cancer cells have been shown to harbor a different metabolic profile than platinum-sensitive ovarian cancer cells; targeting their metabolic pathways, perhaps by some form of calorie restriction, may yield beneficial results in overcoming their resistance [34].

Our study has a few inherent limitations. Firstly, metabolic pathways are complex and interconnected; the genomic signature we chose to employ is a very simplified model, undoubtedly less intricate and diverse than the true metabolic profile presented by cancers in general and ovarian cancer specifically. Secondly, even though we focused on ovarian tissue models, most studies were performed on animals and trials in human ovarian tissue are scarce. A third point is the multitude of dietary interventions available and the potential different effect each modality may have on the transcriptome. Lastly, our database was lacking information on factors that may have influenced survival, such as smoking status, environmental factors and other comorbidities.

We acknowledge all limitations, particularly that our model represents a gross oversimplification. It’s possible that other proteins could have been added or different proteins chosen. Nevertheless, we attempted to mitigate these shortcomings by selecting a genomic profile that has been unequivocally validated, primarily through trials in ovarian tissue. We also took care to select alterations that were consistently induced by most, if not all, dietary interventions. Lastly, the primary aim of this article is conceptual; namely, to shed light on a frequently overlooked aspect—the potential added benefit of caloric restriction to cancer therapy. The identification of a "non-fasting" genomic signature associated with adverse outcomes in ovarian cancer underscores the importance of considering dietary interventions in patient care and strengthens the increasing evidence that nutritional interventions may improve their prognosis [35]. Additional research is required to explore the impact of different forms of caloric restrictions on the human ovarian tissue and whole blood transcriptome, aiming to elucidate their potential role as supplementary approaches to standard treatment of ovarian cancer. While our research findings are focused on ovarian cancer, the methodology employed suggests the potential to create non-fasting genomic signatures for other specific tumors, extending the implications and applicability of our study.

## Conclusions

This study demonstrates that ovarian cancer tumors expressing a "non-fasting" transcriptional profile are associated with poorer outcomes, including reduced overall survival, increased lymphatic invasion, and a higher prevalence of the mesenchymal subtype. These findings suggest the potential impact of caloric restriction in improving patient survival and treatment response in ovarian cancer.

## Supporting information

S1 Fig Gene expression levels distribution of the non-fasting genomic signature genes. The dataset includes ovarian normal tissues (n = 88) and ovarian tumors (n = 419). Statistical significance was assessed using Wilcoxon-rank-sum test, where ****p<0.0001 indicate the significance levels.
(PDF)

S2 Fig Distribution of the Non-Fasting Genomic Signature in normal tissues and primary tumors; (A) prostate (n = 100, n = 495, respectively), (B) testis (n = 165., n = 148, respectively), (C) breast (n = 179, n = 1092, respectively), and (D) colon (n = 308, n = 288, respectively); **** = p<0.0001, Wilcoxon rank-sum test.
(PDF)

S3 Fig Distribution of the non-fasting genomic signature across different residual disease measurements. The dataset includes stage IIIc and stage IV ovarian tumors (n = 383, n = 74, respectively), with information regarding degree of residual disease. Statistical significance was assessed using Kruskal-Wallis sum test, where *p<0.05 indicate the significance levels.
(PDF)

S4 Fig Forest plot representing the results of a multivariable Cox proportional hazards regression model analyzing the association of age, stage and non-fasting genomic signature group with the two years survival outcomes. The hazards ratios (HRs) for each variable are displayed along with their 95% confidence intervals (CIs).
(PDF)

S1 File (XLSX)
