# Supplementary figures and images for: Unlocking prognostic potential: A genomic signature of caloric restriction in patients with epithelial ovarian cancer
Source: PLoS One. 2025 Jan 16;20(1):e0317502. doi: 10.1371/journal.pone.0317502 (PMC11737700; doi:10.1371/journal.pone.0317502)

Gene Expression

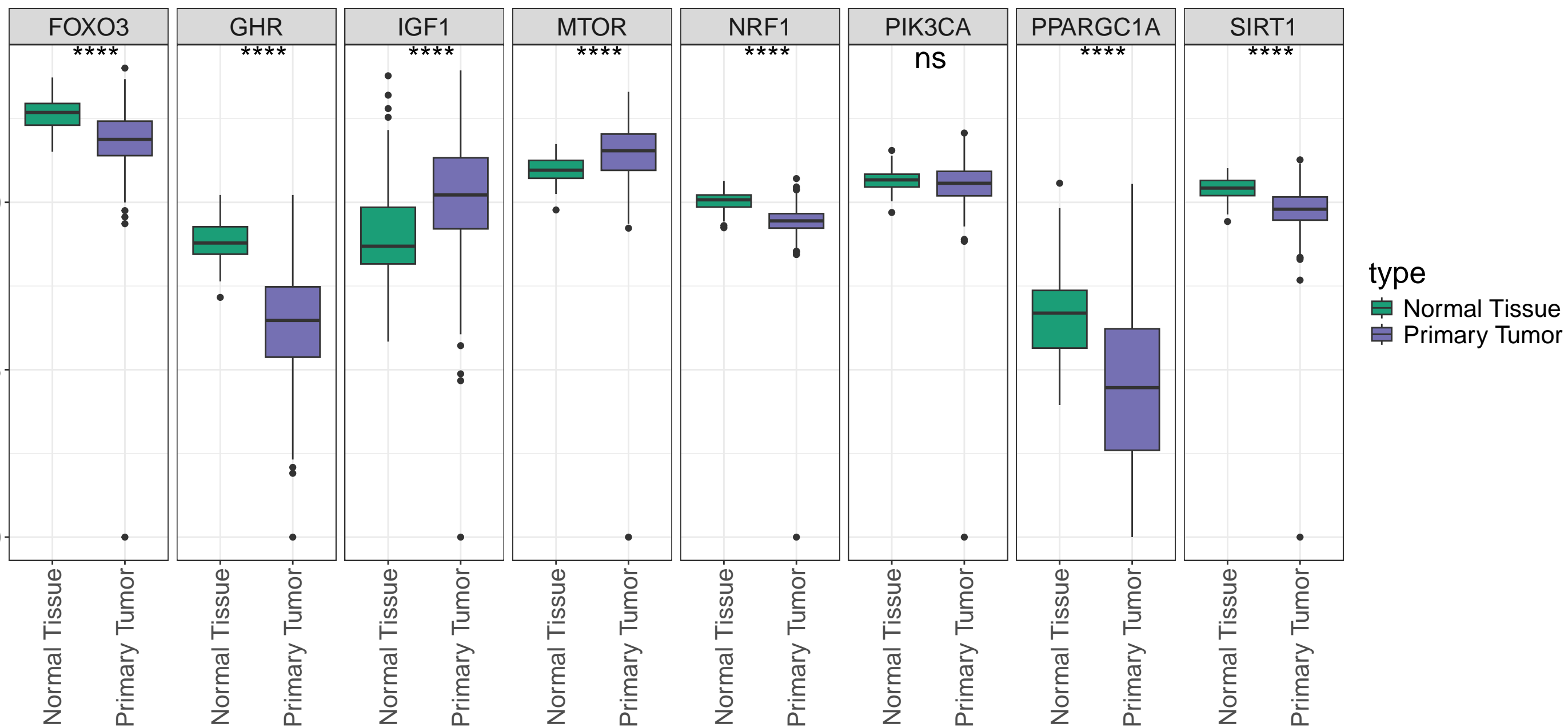

Supplement: S1 Fig — The dataset includes ovarian normal tissues (n = 88) and ovarian tumors (n = 419). Statistical significance was assessed using Wilcoxon-rank-sum test, where ****p<0.0001 indicate the significance levels. (PDF) [file pone.0317502.s001.pdf]

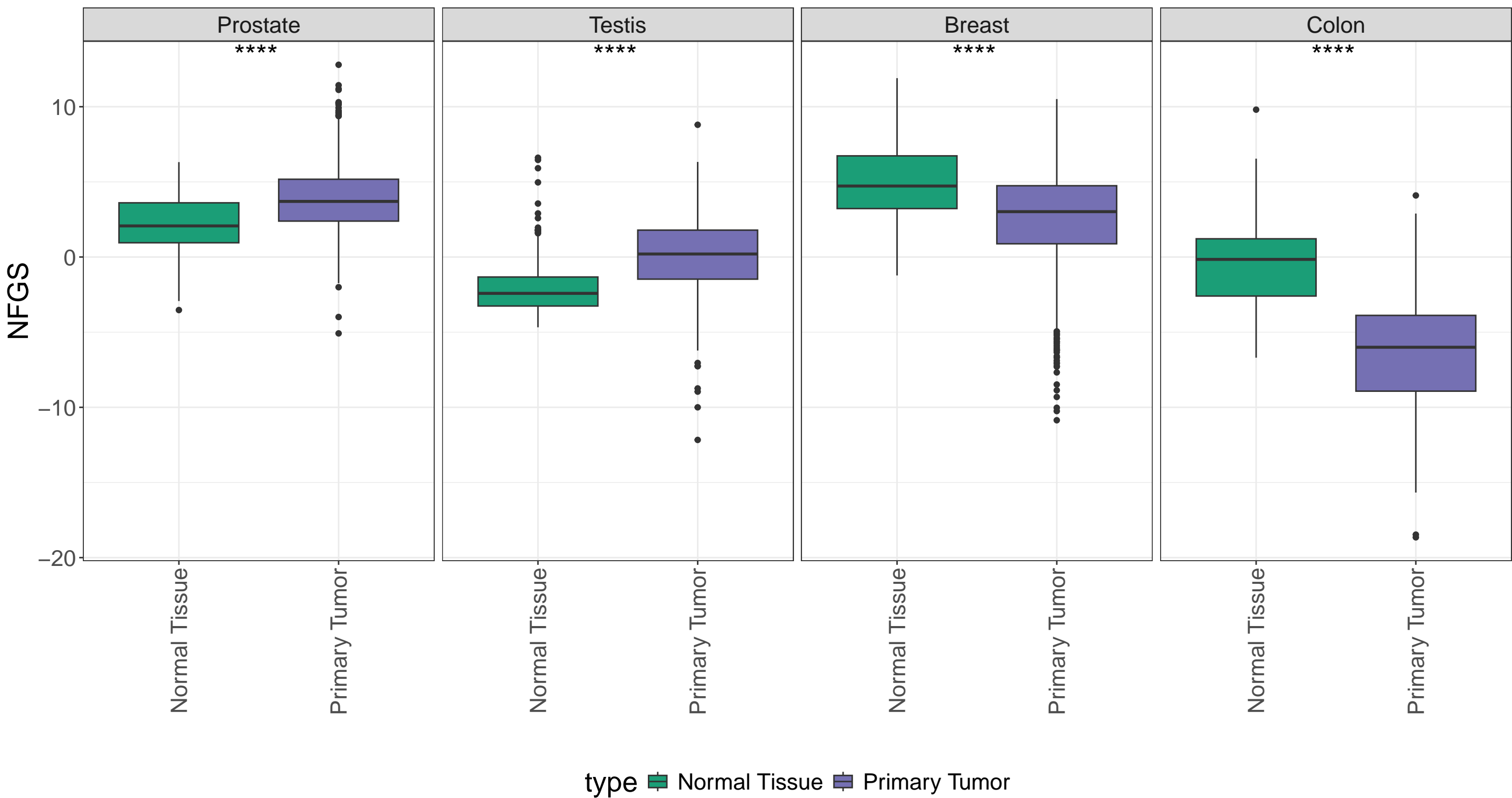

Supplement: S2 Fig — Distribution of the Non-Fasting Genomic Signature in normal tissues and primary tumors; (A) prostate (n = 100, n = 495, respectively), (B) testis (n = 165., n = 148, respectively), (C) breast (n = 179, n = 1092, respectively), and (D) colon (n = 308, n = 288, respectively); **** = p<0.0001, Wilcoxon rank-sum test. (PDF) [file pone.0317502.s002.pdf]

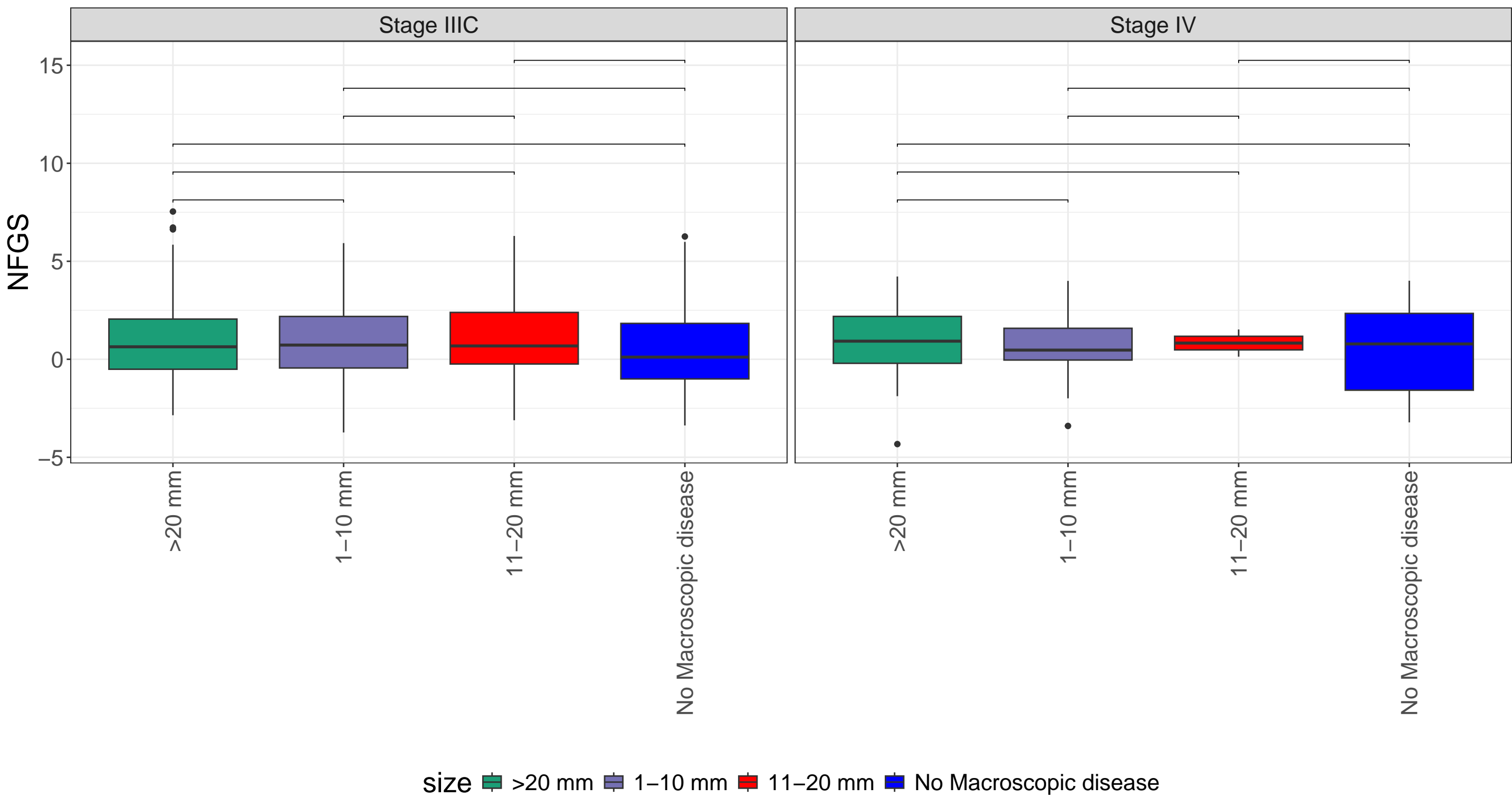

Supplement: S3 Fig — The dataset includes stage IIIc and stage IV ovarian tumors (n = 383, n = 74, respectively), with information regarding degree of residual disease. Statistical significance was assessed using Kruskal-Wallis sum test, where *p<0.05 indicate the significance levels. (PDF) [file pone.0317502.s003.pdf]

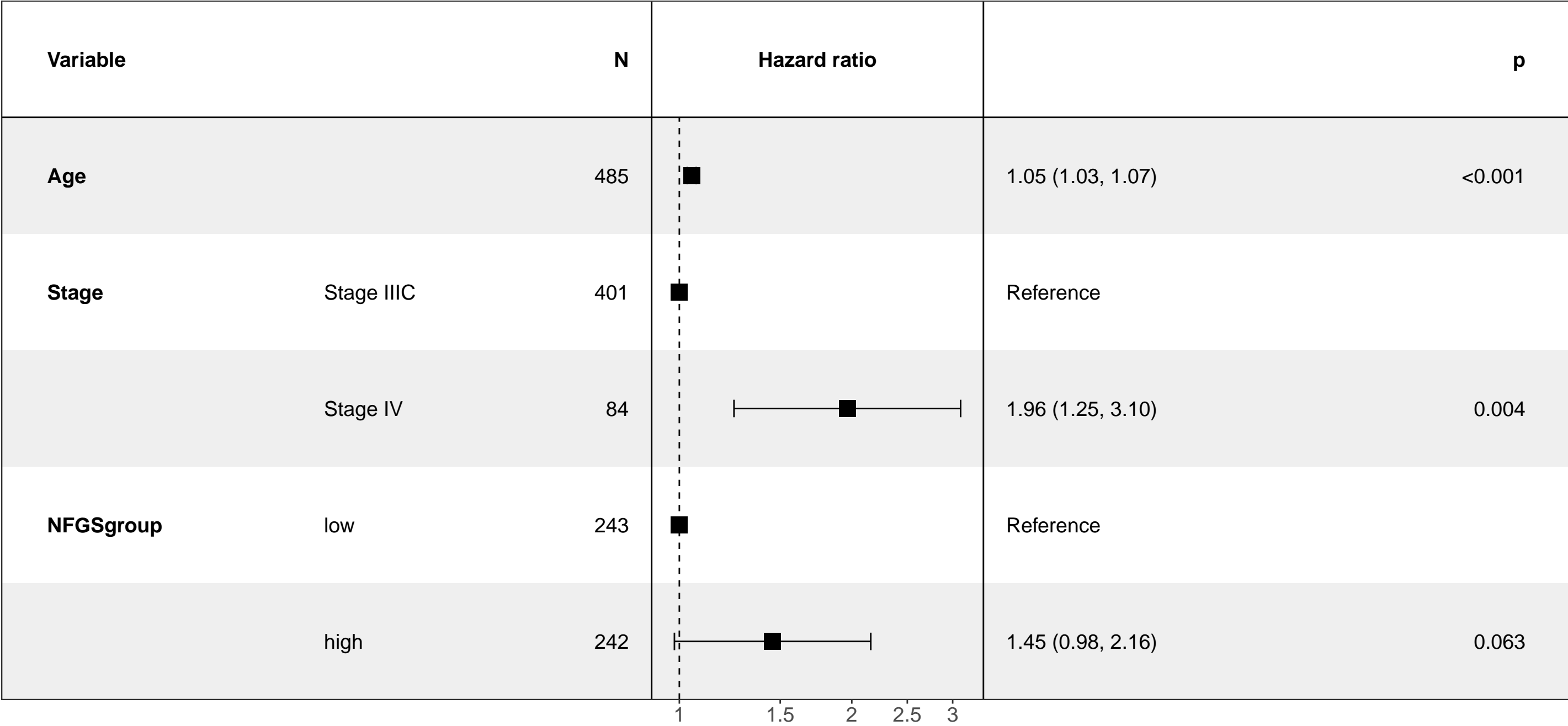

Supplement: S4 Fig — The hazards ratios (HRs) for each variable are displayed along with their 95% confidence intervals (CIs). (PDF) [file pone.0317502.s004.pdf]
